# Comparative effect of feeding Tifton 85 hay and Pornunça hay as roughage on the performance, carcass characteristics, and meat quality of feedlot lambs

**DOI:** 10.1007/s11250-026-05347-5

**Published:** 2026-09-21

**Authors:** Maria Cláudia Soares Cruz Coelho, Carla Wanderley Mattos, Gherman Garcia Leal de Araújo, Kelly Cristina dos Santos, Matheus Henrique Andrade de Silva, João Paulo Ismério dos Santos Monnerat, Luciana Felizardo Pereira Soares, Andreia Fernandes de Souza, Maria Alyne Coutinho Santos, Dorgival Morais de Lima Júnior, Francisco Fernando Ramos de Carvalho

**Affiliations:** 1 Campus Petrolina, Federal Institute of Education, Science and Technology of the Sertão Pernambucano (IFSertãoPE), Aristarco Lopes Street, Center, Petrolina, PE 56302-100 Brazil; 2 Brazilian Agricultural Research Corporation, Semi-Arid EMBRAPA, Highway 428, Petrolina, PE 56302970 Brazil; 3 Department of Animal Science, Federal Rural University of Pernambuco, Dom Manuel de Medeiros Street, Dois Irmãos, Recife, PE 52171-900 Brazil; 4 Department of Animal Science, Federal Rural University of the Semi-Arid, Francisco Mota Street, Costa e Silva, Mossoró, RN 59625-900 Brazil

**Keywords:** Carcass traits, Feedlot, Grass hay, Growth performance, Meat quality

## Abstract

This study aimed to evaluate the effect of replacing Tifton 85 hay (TH) with Pornunça hay (PH) on intake, nutrient digestibility, performance, carcass characteristics, centesimal composition, and physicochemical parameters of the meat of feedlot lambs. We used 36 Santa Inês male lambs, with an age of 4 months and an initial body weight of 18.60 kg ± 3.80 kg, confined for 71 days, with 15 days of adaptation and 56 days of data collection. The animals were distributed in a completely randomized design in four treatments. Dietary treatments consisted of replacing TH with PH at increasing levels (0%, 33%, 67%, and 100%) within the dry matter of the roughage fraction, maintaining a 40:60 roughage-to-concentrate ratio in the total mixed ration. Dry matter (DM), organic matter, and non-fibrous carbohydrates intake increased (*P* < 0.01) by replacing TH with PH. However, there were decreases (*P* < 0.01) in the crude protein and neutral detergent insoluble fiber digestibility coefficients of the diet with increasing PH levels. The final weight, daily gain, and feed conversion of the lambs were 30.96 kg, 0.26 kg/day, and 4.38 kg/kg, respectively; the increase in PH in the diet did not affect (*P* > 0.05) these values. Conversely, hot and cold carcass weights and yields increased (*P* < 0.01) with the dietary replacement of TH by PH. In contrast, TH replacement did not affect (*P* > 0.05) the regional carcass composition of the lambs. Furthermore, the substitution of TH with PH had no impact (*P* > 0.05) on meat color, water-holding capacity, tenderness, or chemical composition (i.e., ash, protein, and fat contents). We recommend completely replacing Tifton 85 hay with Pornunça hay in the diet of feedlot lambs.

## Introduction

Ensuring forage support for the sheep herd in tropical areas is the greatest challenge for production systems installed in these locations (Fernandez-Turren et al. 2020). In addition, climate change can intensify drought events and reduce climate predictability. These challenges only contribute to the vulnerability of production systems conducted in tropical areas, especially in arid and semi-arid localities (Anuta et al. 2025; Nontu et al. 2026). In this context, forage plants that are tolerant to drought, productive, and with high adaptive capacity to environmental hazards (i.e., cuts in different frequencies and intensities) seem to be promising alternatives for feeding animals reared in tropical areas (Souza et al. 2019; Brunetti et al. 2026). Some plants of the *Manihot* genus (*Euphorbiaceae*) meet some of these characteristics (Duputié et al. 2011; Simon et al. 2022).

The Pornunça (*Manihot* spp.) is a natural hybrid between the Manihot (*Manihot pseudoglaziovii* Mull.), a shrub native to tropical areas of South America, and the Cassava (*Manihot esculenta* Crantz), a domesticated plant with high socioeconomic importance (Bredeson et al. 2016; Gomes et al. 2022; Otekunrin et al. 2024). Pornunça shows high phenotypic plasticity, drought resistance, and high regrowth capacity (Alencar et al. 2015). Nevertheless, the presence of cyanogenic glycosides is a challenge for adding forage value to *Manihot* foliage (Soto-Blanco and Górniak 2010). Admittedly, haying reduces the cyanide levels present in Manihot, establishing Manihot hay as a forage resource for small ruminants (Amorim et al. 2005). There is evidence that hay from the aerial part of Cassava or Manihot can replace hay from grasses in the diet of sheep, without negatively affecting performance or carcass characteristics (Moura et al. 2020; Lima et al. 2022).

Specifically, the Pornunça forage has about 155.7 ± 10.0 g/kg of crude protein, 464.4 ± 132.1 g/kg of neutral detergent insoluble fiber, and 261.3 ± 136.8 g/kg of non-fibrous carbohydrates, which place this shrub as a potential forage resource (Viana et al. 2025). In addition, the in vitro ruminal degradability of Pornunça silage (Gomes et al. 2023) and the favorable nutritional effects of the addition of Pornunça in mixed silages (Silva et al. 2023a, b; Godoi et al. 2024) are evidence that strengthens the use of Pornunça as a forage alternative for ruminants. Amorim et al. (2022) tested the total replacement of Tifton 85 hay with Pornunça silage for lambs and verified that Pornunça silage improved the intake and digestibility of nutrients from the diet, without interfering with the physiological parameters of the animals. However, studies evaluating Pornunça hay in the diet of lambs and its effects on performance, carcass characteristics, and meat quality of animals are rare.

Native forage grasses from tropical regions are utilized due to the challenges of cultivating exotic species, such as Tifton or Bermuda grass (*Cynodon* spp.), which typically demand high soil fertility and water inputs (Sanches et al. 2017). We hypothesize that replacing grass hay (Tifton 85) with Pornunça hay improves the performance, carcass characteristics, and meat quality of feedlot lambs. Therefore, this study aimed to evaluate the effect of total replacement (0%, 33%, 67%, and 100%) of Tifton 85 (*Cynodon* spp.) with Pornunça (*Manihot* spp.) hay on the intake, digestibility, performance, carcass characteristics, centesimal composition, and physicochemical parameters of the meat of feedlot lambs.

## Materials and methods

All procedures were conducted following the guidelines set by the the Ethics Committee on the Use of Animals (CEUA) of the *Instituto Federal de Educação*,* Ciência e Tecnologia do Sertão Pernambucano* (CEUA/IF SERTÃO-PE) (License 005/2014).

### Location, animals, and treatments

The experiment was developed at the *Instituto Federal de Educação*,* Ciência e Tecnologia do Sertão Pernambucano* - Petrolina Campus, located in the municipality of Petrolina, in the semiarid region of the state of Pernambuco (Latitude 09° 20′ 19″ S and Longitude 40° 42′ 01″ W). According to the Koppen classification, the region has a climate type BShw’, with a mean annual rainfall of 450 mm. During the experimental period, the maximum and minimum temperatures were 34.5 °C and 21.4 °C, respectively; precipitation was 210.8 mm; and relative humidity was 59.33%.

We used 36 Santa Inês lambs, not castrated, with a mean age of four months and a mean initial body weight of 18.60 kg ± 3.80 kg, housed in 1.0 × 1.5 m individual stalls, shaded with polyethylene covers, with a beaten floor, and provided with a feeder and drinker. The experiment lasted 71 days, with 15 days of animal adaptation to the facilities and diets and 56 days of data collection. Before beginning the experiment, the animals were dewormed, identified, and randomly distributed in individual stalls.

The animals were distributed in a completely randomized design, in four treatments (0, 33, 67, and 100%) of Tifton 85 hay (*Cynodon* spp.) replaced with Pornunça (*Manihot* spp.) hay in the dry matter of the diet. The rations were adjusted to meet the NRC (2007) recommendations for growing lambs, with a mean weight gain of 250 g/day. The ingredients were Tifton 85 hay, Pornunça hay, corn grain, soybean meal, calcitic limestone, and mineral mixture, ensuring a roughage: concentrate ratio of 40:60 (Tables 1 and 2).

**Table 1:** Chemical composition of experimental diet (g/kg of dry matter)

	Ingredients			
	Pornunça hay	Tifton 85 hay	Soybean meal	Corn grain
Dry matter	890.5	887.4	910.9	874.5
Organic matter	931.0	923.1	931.3	983.1
Ash	68.9	76.9	68.7	16.9
Crude protein	134.3	121.3	513.4	91.0
NDIN^1^	25.2	14.7	42.7	10.9
ADIN^2^	13.2	4.90	8.7	41.4
Etheral extract	30.3	16.4	14.5	49.3
NDFap^4^	376.3	705.2	145.7	108.4
ADF^5^	315.9	377.7	92.6	48.7
Lignin	122.8	44.5	13.7	17.9
NFC^6^	390.2	80.2	257.8	734.4

^1^NDIN = Neutral detergent insoluble nitrogen; ^2^ADIN = Acid detergent insoluble nitrogen; ^3^NDF = Neutral detergent insoluble fiber; ^4^NDFap = neutral detergent insoluble fiber corrected for ash and protein; ^5^ADF = Acid detergent insoluble fiber; ^6^NFC = non-fibrous carbohydrates

**Table 2:** Chemical composition of experimental ration

	Levels of replacement of Tifton 85 hay for Pornunça hay (%)			
	0	33	67	100
Pornunça hay	0.0	13.3	26.7	40.0
Tifton 85 hay	40.0	26.7	13.3	0.0
Corn grain	34.6	36.2	37.6	39.0
Soybean meal	21.2	19.7	18.3	16.9
Mineral mix	0.9	0.9	0.8	0.8
Limestone	1.7	1.6	1.7	1.7
NaCl	1.6	1.6	1.6	1.6
	**Chemical composition (g/kg DM)**			
Dry matter	892.1	891.9	891.8	891.7
Organic matter	909.7	912.4	914.2	915.9
Ash	90.3	87.6	85.8	84.0
Crude protein	188.8	184.2	180.1	176.0
Ether extract	26.7	29.1	31.4	33.8
NDFap^1^	350.5	306.3	261.7	217.4
ADF^2^	187.6	178.7	169.8	161.0
Lignin	26.9	37.3	47.9	58.4
NFC^3^	340.8	389.9	438.2	486.1
ME^4^ (Mcal/kg MS)	2.6	2.6	2.6	2.6
TDN ^5^	678.8	660.2	685.1	688.1

^1^NDFap = neutral detergent insoluble fiber corrected for ash and protein; ^2^ADF = Acid detergent insoluble fiber; ^3^NFC = non-fibrous carbohydrates; ^4^ME = Metabolizable energy; ^5^TDN = Total digestible nutrients

The Tifton 85 hay and Pornunça hay were crushed in a machine with a 12 mm sieve. The corn grain and soybean meal were crushed in a machine with a 5 mm sieve. The diets were offered as a complete ration in two meals per day at 09 h:00 min and 15 h:00 min. During the data collection period, the feed provided and the leftovers were weighed and recorded daily to calculate intake, adjust the feed offered, and ensure 15% leftovers in relation to the intake of the day before. The feed offered and the leftovers were sampled weekly and pre-dried in an air-circulation oven at 55 °C for 72 h. Water was provided ad libitum, and intake was quantified once per week during the data collection period.

### Nutrient and water intake and dietary nutrients digestibility

Nutrient intake was determined by quantifying the feed offered and the leftovers, and by analyzing their chemical composition.

The digestibility test began on the 31st day of the experimental period, with 24 lambs randomly selected (six per treatment). The test lasted 7 days, with the first 4 days for animal adaptation to the bags and the last 3 days for recording feed offered, water, leftovers, and feces. Feces were collected twice daily (morning and afternoon) using bags adapted for total fecal collection, adjusted to the animal’s body, totaling three samples per animal. The fecal samples were weighed, placed in a forced-ventilation oven at 55–60 °C for 72 h, and packed in plastic bags. They were later ground in a Willey-type knife mill, with a 1.0 mm sieve, and a representative aliquot was removed from each subsample, homogenized to form a composite sample, packed in polyethylene containers with a screw cap, and stored for bromatological analysis. Apparent digestibility was calculated according to the methodology proposed by Berchielli et al. (2011).

Water intake was measured once per week during the data collection period. Before the supply, the water offered was weighed into plastic containers with a 10-liter capacity; the following day, the leftovers were weighed, and the daily water intake was calculated by subtracting the leftovers and losses by evaporation from the water offered. Evaporation losses were measured from buckets of water distributed at different points of the installation.

### Chemical composition of the ingredients, leftovers, and feces

Ingredient, leftover, and feces samples were analyzed for dry matter (DM, method 934.01), organic matter (OM, method 930.05), ash (method 942.05), crude protein (CP, N Kjeldahl × 6.25, method 981.10), and ether extract (EE, method 920.39) according to the AOAC (2000) procedures. The neutral detergent insoluble fiber (NDF) content was determined as described by Van Soest et al. (1991): thermostable alpha-amylase was used, and the residue was corrected for ash (Mertens 2002) and protein (Licitra et al. 1996) (NDFap). Neutral detergent-insoluble nitrogen (NDIN) and acid detergent-insoluble nitrogen (ADIN) were determined according to Licitra et al. (1996). The total carbohydrate content (TCC) was calculated using the equation proposed by Sniffen et al. (1992), and non-fibrous carbohydrates (NFC) were calculated according to Hall (2003). The equation described by Weiss (1999) was used to estimate total digestible nutrient (TDN) concentration. The analyses were performed in the Animal Nutrition Laboratories of EMBRAPA – Petrolina, Brazil.

Nutrient digestibility coefficients were obtained by the difference between the amount of feed offered and the amount ingested. The total digestible nutrient intake (TDNI) was obtained according to Sniffen et al. (1992). Digestible energy (DE) was calculated as the product of NDT content and factor 4409, and ME concentration was considered 82% of DE (Sniffen et al. 1992).

### Performance and carcass characteristics

For the performance test, at the end of the adaptation phase, the initial body weight (BWi) of the animals was recorded before supplying the feed and without fasting for solids or water. Subsequent weighings (Scale, BL300SDIG, Laboremus, Brazil) were performed every 14 days until the end of the experimental period, when the animals were weighed to obtain the final body weight (BWf) and transported to the municipal slaughterhouse in Petrolina, PE.

Animal performance was evaluated using the daily weight gain (DWG), feed conversion (FC), and water intake. Total weight gain (TWG) was calculated as the difference between BWf and BWi, and the average daily gain (ADG) was calculated from the relationship between TWG and days regarding the performance period (56 days). Feed conversion (FC) was calculated from the dry matter intake (DMI) in relation to ADG (FC = DMI / ADG).

Regarding the slaughter procedure and carcass evaluations, at the end of the confinement period and after obtaining the final body weight (BWf), the animals were transported to the municipal slaughterhouse in Petrolina, PE. After fasting from solids for 17 h, the animals were weighed to record body weight at slaughter (BWS) and then slaughtered in accordance with Brazilian regulations (Brasil, 2000). Slaughter was performed by stunning non-penetrating percussion, followed by bleeding with carotid and jugular incision. After skinning and evisceration, the head (section in the atlanto-occipital joint) and legs (section in the carpal and tarsometatarsal joints) were removed to record the hot carcass weight (HCW).

Weight loss resulting from imposed fasting was obtained by the expression FW (%) = {(BWf – BWS) ÷ (BWf) x 100}. The hot carcasses were weighed, determining the hot carcass yield using the formula HCY (%) = {(HCW ÷ BWS) x 100}, and taken to the cold chamber for 24 h, at 4 °C, with the tarsometatarsal joints distanced by hooks. After the 24 h period, the linear measurements taken on the carcasses were noted according to Cezar and Sousa (2007). Carcass compactness indices (CCI (kg/cm) = cold carcass weight ÷ carcass internal length) and leg compactness indices (LCI (kg/cm) = leg weight ÷ leg length) were calculated. Subsequently, the cold carcass weight (CCW) was recorded, obtaining the cooling loss CL (%) = {(HCW-CCW) ÷ HCW} x100 and cold or commercial carcass yield (CY (%) = {(CCW ÷ BWS) x 100}.

After removing the tails, the carcasses were divided longitudinally at the midline into two forequarters, and the left half-carcass was sectioned into six anatomical regions, according to methodologies adapted from Cezar and Sousa (2007): neck, rib, shoulder, loin, leg, and hindquarter. The individual weight of each cut was recorded immediately after its removal from the carcass. Subsequently, the weight of the six cuts was used to determine the weight of the reconstituted cold half-carcass (WRCHC) and the commercial yield of the carcass cuts. The loin of each lamb was vacuum-packed and immediately frozen for further analysis.

A cross-section was performed in the dorsal portion of the *Longissimus dorsi* muscle, between the 12th and 13th thoracic vertebrae, and measurements were taken to calculate the rib eye area (REA), according to Cezar and Sousa (2007). The measurements were taken using a caliper and consisted of: maximum muscle length (measure A), maximum muscle depth (B), minimum thickness of the covering fat on the muscle (C), and maximum thickness of covering fat on the surface of the 12th rib, 11 cm from the dorso-lumbar line (GR - grade rule). The REA was then calculated using the equation: REA = (A/2 x B/2) π, with π equal to 3.1416.

### Centesimal composition and physicochemical characteristics of the meat

The left loin of each lamb was thawed at 4 °C, and the *Longissimus dorsi* muscle was removed, and the physicochemical analyses and centesimal composition of the meat were immediately performed. Chemical composition (moisture, crude protein (CP), mineral matter (MM), and ether extract (EE) was analyzed according to AOAC (2000) procedures: methods 930.15, 942.05, 984.13, and 920.39, respectively.

After the samples were exposed to air in a refrigerated environment (4 ± 1 °C) for 30 min to allow the surface oxygenation of myoglobin in the muscle, readings were performed with a colorimeter to characterize meat color. Three measurements were performed at different points of the muscle, noting the values of L*, a*, and b*, where L* indicates the luminosity, a* indicates the red color and b* indicates the intensity of the yellow color (Ramos and Gomide 2009).

Water retention capacity (WCR) was determined according to Sierra (1973). A total of 300 mg of meat was placed between two previously weighed circular filter papers for weighing. The sample was then placed between two inverted Petri dishes and subjected to a 3.4 kg weight for 5 min. After this time, the sample was removed immediately, and the filter paper (W) was weighed. The difference between the final and initial weights represents the juice expelled by the meat sample and was calculated using the following formula: WRC (%) = (Wfinal – Winitial)/A x 100. Where “A” represents the weight of the sample in g.

Cooking losses were quantified in 2.5 cm-thick samples wrapped in aluminum foil and baked in a preheated conventional oven at 200 °C. The samples were removed from the oven after the temperature in the geometric center reached 70 °C and cooled at room temperature. Cooking losses were calculated as the difference in the sample’s weight before and after cooking, expressed as a percentage (Duckett et al. 1998).

After cooking, four cylindrical samples were taken in the longitudinal direction of the meat fiber, proceeding to the objective analysis of the texture (shear force), using the Warner-Bratzler Shear Force equipment (G-R MANUFACTURING CO, Model 3000) with a load cell of 25 kgf and a speed of 20 cm/min (Duckett et al. 1998).

### Statistical analysis

The data were submitted to the Bartlett test to verify homoscedasticity and the Shapiro-Wilk test to verify normality. Once the premises were satisfied, the data were submitted to analysis of variance (ANOVA) using the GLM procedure of the Statistical Analysis System - SAS software (version 9.4), according to the following model:

Yij = µ + Ti + β (Xij − X) + eij;

Where: Yij = observed value of the dependent variable; µ = general mean; Ti = effect of treatment i (i = 0, 33, 67, and 100% of replacement); β (Xij - X) = covariate effect (initial weight was used as a covariable); eij = experimental error.

For regression analysis (PROC REG), the sum of the squares of the treatments was decomposed into two contrasts: linear (− 3–1 + 1 + 3) and quadratic (+ 1 − 1–1 + 1), at a significance level of 5% (*P* < 0.05).

## Results

Dry matter (DM), organic matter (OM), and non-fibrous carbohydrates (NFC) intake increased (*P* < 0.01) by replacing Tifton 85 hay (TH) with Pornunça hay (PH). On the other hand, there was a decrease (*P* < 0.01) in the neutral detergent insoluble fiber (NDFap) intake of the animals with an increase in the proportion of PH in the diet. The replacement of TH with PH did not affect (*P* > 0.05) crude protein (CP) and total digestible nutrients (TDN) intake (Table 3).

**Table 3:** Intake and apparent digestibility of nutrients from the diet of lambs receiving Pornunça (*Manihot* spp.) hay replacing Tifton 85 hay

Intake (kg/dia)	Levels of replacement of Tifton 85 hay for Pornunça hay (%)				SEM^1^	*P* value^2^	
	0	33	67	100		L	Q
Water	3.16	3.25	3.40	3.50	0.098	0.7108	0.9242
Dry matter	1.06	1.10	1.18	1.23	0.025	0.0060^a^	0.7608
Organic matter	0.96	1.00	1.08	1.13	0.024	0.0040^b^	0.6970
Crude protein	0.22	0.22	0.22	0.23	0.005	0.8734	0.4021
Ether extract	0.03	0.04	0.04	0.04	0.001	< 0.0001^c^	0.3758
NDFap^3^	0.32	0.30	0.29	0.24	0.008	< 0.0001^d^	0.5412
NFC^4^	0.40	0.45	0.54	0.63	0.017	< 0.0001^e^	0.2203
TDN^5^	0.77	0.74	0.76	0.95	0.039	0.7608	0.4204
**Digestibility (%)**							
Dry matter	71.64	70.36	71.23	71.29	0.762	0.4978	0.9259
Organic matter	71.90	71.04	71.99	72.14	0.736	0.1102	0.7148
Crude protein	75.31	72.31	71.03	65.96	0.911	0.0020^f^	0.9684
Ether extract	83.55	73.17	71.99	65.99	2.354	0.0120^g^	0.3900
NDFap^3^	60.65	42.19	39.64	31.29	2.432	< 0.0001^h^	0.5903
NFC^4^	77.74	82.10	85.39	88.41	1.243	< 0.0001^i^	0.2577

^1^SEM = standard error of the mean; ^2^P value: L = Linear and Q = Quadratic; ^3^NDFap = neutral detergent insoluble fiber corrected for ash and protein; ^4^NFC = non-fibrous carbohydrates; ^5^TDN = total digestible nutrients. Equations: ^a^Ŷ = 988.2194 + 61.0609X, r^2^ = 0.98; ^b^Ŷ = 888.7394 + 60.3282X, r^2^ = 0.98; ^c^Ŷ = 27.11617 + 4.3742X, r^2^ = 0.99; ^d^Ŷ = 347.2444–23.7541X, r^2^ = 0.92; ^e^Ŷ = 304,5711 + 80,4080X, r^2^ = 0.99; ^f^Ŷ = 78.4800–2.9315X, r^2^ = 0.71; ^g^Ŷ = 87.1425–5.3878X, r^2^ = 0.91; ^h^Ŷ = 66.0975–9.0622X, r^2^ = 0.90; ^i^Ŷ = 74.5883 + 3.5285X, r^2^ = 0.99

The DM and OM apparent digestibility coefficients of the lambs’ diet were 71.24% and 71.87%, respectively; replacing TH with PH did not affect (*P* > 0.05) these values. However, there were decreases (*P* < 0.01) in the CP and NDFap digestibility coefficients of the diet with increasing PH levels. Additionally, we observed an increase in the apparent digestibility coefficient of the diet’s NFC when TH was replaced with PH.

The final weight, average daily gain, and feed conversion of the lambs were 30.96 kg, 0.26 kg/day, and 4.38 kg/kg, respectively; the increase in PH in the diet did not affect (*P* > 0.05) these values. However, the weights and yields of hot and cold carcasses increased (*P* < 0.01) by replacing the TH with PH in the diet. We observed no effect (*P* > 0.05) of replacing TH with PH for cooling losses, compactness index, rib eye area, or subcutaneous fat thickness of the lambs’ carcass (Table 4).

**Table 4:** Performance and carcass characteristics of lambs receiving Pornunça (*Manihot* spp.) hay replacing Tifton 85 hay

Variables	Levels of replacement of Tifton 85 hay for Pornunça hay (%)				SEM^1^	*P* value^2^	
	0	33	67	100		L	Q
Initial weight (kg)	18.20	18.67	18.87	18.53	0.286	0.1837	0.4522
Final weight (kg)	32.40	32.69	33.56	34.24	0.506	0.4553	0.2076
Body weight at slaughter (kg)	30.07	30.40	31.51	31.87	0.446	0.1060	0.8571
Average daily gain (kg/day)	0.25	0.25	0.26	0.28	0.006	0.5671	0.3927
Feed conversion (kg/kg)	4.20	4.41	4.49	4.44	0.067	0.4292	0.4049
Hot carcass weight (kg)	14.07	14.08	15.21	15.58	0.234	0.0050^a^	0.7306
Cold carcass weight (kg)	13.78	13.77	14.91	15.27	0.231	0.0050^b^	0.4987
Cooling losses (%)	2.07	2.23	2.01	2.00	0.049	0.1891	0.9616
Hot carcass yield (%)	46.80	46.36	48.27	48.93	0.266	< 0.0001^c^	0.0560
Cold carcass yield (%)	45.83	45.33	47.30	47.95	0.271	< 0.0001^d^	0.2433
Leg compactness index (kg/cm)	0.27	0.27	0.29	0.30	0.001	< 0.0001^e^	0.6428
Carcass compactness index (kg/cm)	0.06	0.06	0.06	0.06	0.004	0.1296	0.4782
Rib eye area (cm^2^)	12.87	12.15	13.57	13.02	0.240	0.2230	0.4522
Subcutaneous fat thickness (mm)	2.08	2.11	2.72	2.57	0.126	0.3145	0.2076

^1^SEM = standard error of the mean; ^2^*P* value: L = Linear and Q = Quadratic. Equations: ^a^Ŷ = 13.3159 + 0.5679X, r^2^ = 0.89; ^b^Ŷ = 13.0250 + 0.5621X, r^2^ = 0.88; ^c^Ŷ = 45.5128 + 0.8304X, r^2^ = 0.79; ^d^Ŷ = 44.5194 + 0.8330X, r^2^ = 0.78; ^e^Y = 0.2578 + 0.0097X, r^2^ = 0.73

Replacing TH with PH did not affect (*P* > 0.05) the regional composition of the lambs’ carcass, which presented mean proportions of 9.72% of neck, 18.98% of shoulder, 17.34% of ribs, 9.93% of loin, and 33.71% of leg (Table 5).

**Table 5:** Absolute and relative weights of cuts of meat from the carcass of lambs receiving Pornunça (*Manihot* spp.) hay replacing Tifton 85 hay

Variables	Levels of replacement of Tifton 85 hay for Pornunça hay (%)				SEM^1^	*P* value^2^	
	0	33	67	100		L	Q
Neck (kg)	0.64	0.66	0.79	0.75	0.022	0.0030^a^	0.1121
Shoulder (kg)	1.31	1.35	1.40	1.47	0.018	0.3599	0.3393
Ribs (kg)	1.21	1.17	1.33	1.34	0.028	0.0818	0.1247
Loin (kg)	0.66	0.72	0.74	0.77	0.071	0.8373	0.4789
Leg (kg)	2.38	2.34	2.52	2.57	0.039	0.5783	0.6389
Hindquarter (kg)	0.66	0.72	0.73	0.74	0.018	0.7647	0.4235
Neck (%)	9.31	9.28	10.48	9.81	0.124	0.8234	0.8979
Shoulder (%)	19.07	19.00	18.55	19.30	0.158	0.4836	0.4533
Rib (%)	17.69	16.51	17.55	17.63	0.210	0.7242	0.9023
Loin (%)	9.66	10.08	10.24	9.76	0.143	0.7266	0.5678
Leg (%)	34.71	32.93	33.43	33.80	0.218	0.2222	0.1121
Hindquarter (%)	9.56	10.33	9.75	9.70	0.199	0.3599	0.3393

^1^SEM = standard error of the mean; ^2^*P* value: L = Linear and Q = Quadratic. Equations: ^a^Ŷ = 0.5958 + 0.0454X, r^2^ = 0.64

Replacing TH with PH did not affect (*P* > 0.05) the chemical composition (i.e., ash, protein, and fat contents), color, water retention, or tenderness of the meat (Table 6).

**Table 6:** Centesimal composition and physicochemical characteristics of meat (*Longissimus lumborum*) of lambs receiving Pornunça (*Manihot* spp.) hay replacing Tifton 85 hay

Variables	Levels of replacement of Tifton 85 hay for Pornunça hay (%)				SEM^1^	*P* value^2^	
	0	33	67	100		L	Q
Moisture (%)	74.84	74.80	74.91	74.61	0.064	0.8041	0.6468
Ash (%)	1.36	1.43	1.43	1.35	0.021	0.3599	0.2379
Crude protein (%)	21.11	21.36	21.02	21.19	0.072	0.9095	0.3631
Crude fat (%)	2.51	2.61	2.76	2.56	0.054	0.3480	0.3882
pH24h	5.97	5.84	5.86	5.94	0.031	0.9012	0.4057
Luminosity (L*)	37.78	39.39	37.72	35.23	0.859	0.7972	0.4503
Red content (a*)	14.46	14.37	13.42	14.38	0.247	0.6233	0.2904
Yellow content (b*)	13.16	13.11	11.45	12.57	0.397	0.2315	0.0675
Cooking losses (%)	33.31	27.46	29.68	31.80	1.274	0.2957	0.1806
Water retaining capacity (%)	33.21	33.72	34.07	34.15	0.214	0.2370	0.1053
Shear force (kgf/cm^2^)	1.84	1.99	2.18	2.08	0.072	0.9170	0.2402

^1^SEM = standard error of the mean; ^2^*P* value: L = Linear and Q = Quadratic

## Discussion

The Pornunça hay (PH) presented lower levels of NDFap (376.3 g/kg vs. 705.2 g/kg) and higher levels of EE (30.3 g/kg vs. 16.4 g/kg), neutral detergent insoluble nitrogen (NDNI) (25.2 g/kg vs. 14.7 g/kg), NFC (390.2 g/kg vs. 80.2 g/kg), and lignin (122.8 g/kg vs. g/kg vs. 44.5 g / kg) than the Tifton 85 hay (TH).

The increases in DM and OM intake observed can be partially attributed to the lower levels of NDFap and higher levels of NFC in the treatment diets, resulting from increased replacement of TH with PH. Pornunça hay may contain more digestible fractions of carbohydrates (less NDFap and more NFC), resulting in greater degradability of ruminal organic matter and faster disappearance of digesta (Lima et al. 2022; Godoi et al. 2024), favoring increases in animal intake. In addition, the lower NDFap intake observed in lambs, with increased PH in the diet, may have reduced ruminal filling and favored the higher intake of DM of animals with higher levels of PH (Galyon et al. 2024). Consistently, Lima Júnior et al. (2014) and Amorim et al. (2022) also reported an increase in DM intake of lambs when grass hay was replaced with Pornunça silage or *Manihot* hay (*Manihot glaziovii*), respectively.

There was a decrease in the apparent digestibility of dietary CP of lambs with the increase in PH in their diet, possibly due to slow or no availability of the protein fraction of Pornunça hay (25.2 g/kg vs. 14.7 g/kg of NDIN in DM and 13.2 g/kg vs. 4.9 g / kg of insoluble nitrogen in acid detergent in DM) in relation to Tifton 85 hay (Gomes et al. 2023). In this context, Silva et al. (2023a, b) reported that Pornunça silage led to greater fecal nitrogen losses in lambs, thereby reducing dietary CP digestibility. The reduced CP digestibility observed with increasing levels of PH substitution for grass hay may be attributed to the higher concentration of secondary compounds, such as tannins, flavonoids, and saponins, present in PH (Silva et al. 2023a, b). Santos et al. (2021) also reported a decrease in CP digestibility when the *Manihot* hay (*Manihot glaziovii*) replaced TH in the diet of sheep.

We observed a decrease in NDFap digestibility with the replacement of TH with PH, possibly due to the characteristics of the Pornunça fiber. The PH cell wall is more lignified (about 176% more than TH), which explains the lower NFDap degradation with increased PH in the diet (Raffrenato et al. 2017). In addition, the presence of tannins in Pornunça may have compromised the digestibility of the fibrous fraction due to complexation with microbial extracellular enzymes (Archimede et al. 2016), making it difficult to install the ‘log’ phase of microbial growth in the rumen of lambs (Sun et al. 2025). In this context, another point of attention is the increase in dietary NFC intake and apparent digestibility, which may have altered ruminal pH and microbial dynamics, reducing populations of structural carbohydrate-degrading bacteria (Firkins et al. 2024). Thus, the reduction in the populations and diversity of structural carbohydrate-degrading bacteria may explain the decrease in NDFap digestibility observed with the increased replacement of TH with PH (Souza et al. 2026). Occasionally, the increase in NFC apparent digestibility can be attributed to the increase in the intake of this fraction and the increase in NFC from PH, generally pectin, glucans, and fructosans, more degradable fractions of carbohydrates in forages (Villalba et al. 2021).

The increase in carcass weights observed when replacing TH with PH can be attributed to an improved flow of digestible nutrients, primarily derived from the fermentation and digestion of non-fibrous carbohydrates in the diet (Harmon and Swanson 2020). In this context, the increased synthesis and absorption of short-chain fatty acids—along with some glucose absorption in the intestine—provided a greater abundance of energy substrates (Ye et al. 2026) to support empty body anabolism in the lambs, thereby explaining the higher carcass weights (Zhang et al. 2023). Similarly, Lima et al. (2021) reported that replacing TH with cassava hay (*Manihot esculenta* Crantz) in lamb diets resulted in heavier carcasses. Conversely, replacing TH with PH did not affect slaughter body weight or average daily gain. This lack of effect was possibly due to bias associated with the content of the gastrointestinal tract (CGIT) during body weight measurements. Even after fasting, the CGIT constitutes a considerable portion (~ 12%) of body weight (Jardines-Pozos et al. 2025); as an abiotic component, it is largely influenced by the nature of the diet (Campos et al. 2017a, b; Ribeiro et al. 2023). Thus, it can be speculated that the digesta of lambs fed higher levels of TH remained longer in the rumen, even during fasting (consistent with the higher NDFap intake). Consequently, at the time of weighing, this digesta represented a larger portion of body weight relative to non-carcass constituents. Furthermore, the increases in carcass yields observed with higher PH inclusion levels strengthen the inference that the CGIT biased the animal weight measurements upward (Al-Ghamdi et al. 2025).

The observed increases in both hot and cold carcass yields of lambs resulting from the replacement of TH with PH may be linked to a greater anabolism of carcass constituents—as evidenced by the increase in leg compactness—and greater carcass fat deposition (Li et al. 2025; Ebrahim and Alemayehu, 2024). In this last attribute, although there was no statistical difference for the subcutaneous fat thickness of the carcasses, the increase in the replacement of TH with PH coincided with a numerical increase in the subcutaneous fat thickness. This finding is especially relevant for the Santa Inês genotype, which naturally presents high variation in subcutaneous fat deposition in the carcass (Costa et al. 2017; Paim et al. 2022). Lima Júnior et al. (2014) also reported similarity in subcutaneous fat thickness when TH was replaced with *Manihot* hay in the diet of feedlot lambs.

Despite the increase in cold carcass weight, we observed no differences in the relative weights of the carcass cuts, indicating that the slaughtered animals were at a similar physiological stage, with carcasses presenting similar regional composition and, therefore, equivalent body development (Yalcintan et al. 2025). Moura et al. (2020) also reported similar regional composition for lamb carcasses receiving *Manihot* hay replacing TH. This similarity in the physiological stage at slaughter likely justifies the similarity of the physicochemical characteristics of the meat of lambs (Aksoy et al. 2019).

The replacement of TH with PH did not affect the moisture (74.8%), ash (1.4%), protein (21.2%), or fat (2.6%) contents of the lambs’ meat. Fat content is the component most influenced by diet (Zhang et al. 2022), but considering the weight at maturity of whole males of the Santa Inês genotype, the nutritional differences between the treatment diets were probably not enough to allow differential deposition of triglycerides in the intramuscular adipocytes of the lambs (Fernandes Júnior et al. 2021). Campos et al. (2017a, b) reported values of 74.7% moisture, 1.3% ash, 23.0% protein, and 2.5% fat in the meat of lambs fed with Pornunça silage.

The carcass pH, 24 h post-mortem, was 5.9 and did not vary with the replacement of TH with PH, possibly due to similarity in muscle glycogen stores of lambs at the time of slaughter (Ferguson and Gerrard 2014). We speculate that the observed differences in energy intake and carcass weight of the lambs, with the replacement of TH with PH, were not enough to interfere with the muscle glycogen stores of these animals significantly (Gonzales-Barron et al. 2021). The similarity observed in the final pH values of the carcass helps to explain the equivalence observed for luminosity (37.5 L*), red content (14.2 a*), yellow content (12.6 b*), cooking losses (30.6%), water retaining capacity (33.8%), and shear strength (2.0 kgF/cm^2^) from the meat of lambs (Ding et al. 2024). From the objective measures, we can infer that the meat produced by animals fed PH had a quality equivalent to that produced by lambs fed TH. Sá et al. (2025) reported values (mean ± standard deviation) of 46.6 ± 39.7 L*, 17.5 ± 3.9 a*, 7.7 ± 2.1 b*, 27.6 ± 7.7% for cooking losses, and 3.1 ± 1.0 kgF/cm^2^ for lambs of breeds native to Brazil.

## Conclusion

Replacing Tifton 85 hay with Pornunça hay increased the intake and digestibility of non-fibrous carbohydrates in the diet. It increased carcass weight and yield without compromising lambs’ performance or meat quality. Total replacement of Tifton 85 hay with Pornunça hay is recommended in diets with a roughage: concentrate ratio of 40:60 for feedlot lambs.

Pornunça hay is a drought-resistant forage resource with high nutritional value that can help raise the resilience of sheep meat production systems in tropical regions of the planet.

## Data Availability

Not applicable.
